# Causes of Failure to Capture in Pacemakers and Implantable Cardioverter-defibrillators

**DOI:** 10.19102/icrm.2020.110207

**Published:** 2020-02-15

**Authors:** Ebrahim Sabbagh, Thaer Abdelfattah, Mohammad M. Karim, Amjad Farah, Blair Grubb, Saima Karim

**Affiliations:** ^1^Division of Electrophysiology, Department of Cardiovascular Medicine, University of Toledo Medical Center, Toledo, OH, USA; ^2^Deparment of Internal Medicine, Cleveland Clinic, Cleveland, OH, USA; ^3^Department of Cardiology, Metrohealth Medical Center, Cleveland, OH, USA

**Keywords:** Cardiac implantable electronic devices, implantable cardioverter-defibrillator malfunction, loss of capture, noncapture, pacemaker malfunction

## Abstract

The number of patients with implantable electronic cardiac devices is continuously increasing. As more pacemakers and implantable cardioverter-defibrillators (ICDs) are being placed, a basic understanding of some troubleshooting for devices is becoming essential. Loss of capture can be an emergent presentation for an unstable patient and can be encountered intermittently in hospitalized patients. There are many causes for a loss of capture, with the timing of the implant having a high correlation with certain causes over others. The most common acute cause just after the insertion procedure is lead dislodgement or malposition. In comparison, an increase in the required threshold promoting a loss of capture can happen after months to years of insertion of the pacemaker or ICD. This change can be due to a cardiomyopathy, fibrosis medications, metabolic imbalance, lead fracture, or an exit block. Loss of capture can also occur from external electrical stimuli and inappropriate pacemaker or ICD settings. Further, there are also potential noncardiac causes, such as medications, electrolyte imbalance, and acidemia. A knowledge of these factors is essential for health care providers, given the morbidity and mortality that can potentially be associated with device-related issues, especially in patients who are dependent on the included pacing function.

## Introduction

The number of patients with implantable cardiac devices is continuously increasing.^1,2^ Health-care providers have frequent interactions with patients with pacemakers and implantable cardioverter-defibrillators (ICDs). Therefore, a basic understanding of normal device function, device malfunction, and troubleshooting has become an essential thing to have. There is a frequent need for the evaluation of these devices for the clinical benefit of monitoring the patient’s rhythm abnormalities and events that have occurred, along with the need for therapy.^2,3^ Although it is important to be able to assess arrhythmias and perform device management, physicians should also be aware of device and lead malfunctions and failures.^3,4^ Pacemaker and ICD lead malfunctions can be classified based on the electrocardiogram signs into the following groups: loss of capture, inadequate output, undersensing or oversensing, inappropriate pacing, pacemaker-mediated tachycardia, and issues with battery life. It is common to encounter some of these issues, with failure to capture being an important factor that requires assessment and therapy.^5^

**Figure 1:**
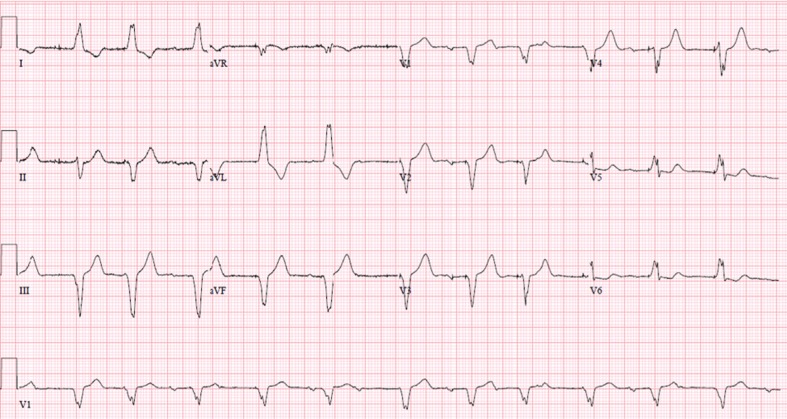
Atrial lead intermittently pacing after undersensing and displaying a loss of capture while the ventricular lead demonstrates appropriate capture upon pacing.

Loss of capture, also known as noncapture, is when the myocardium does not respond to the electrical stimuli from the pacemaker or ICD. On the electrocardiogram or rhythm strip, a pacing spike can be seen with no P or QRS complex subsequently following the pacing spike.^6^ An example is shown in **Figure 1**, where the atrial pacing stimuli do not capture the atrial tissue and, therefore, there is no atrial depolarization with P waves following the pacing stimuli. During the device interrogation, there may be an indication of pacing on the near- or far-field electrocardiogram without an appropriate capture of the chamber being paced. There are many causes for the loss of capture, with the timing of the implant having a high correlation with specific causes (especially immediately postimplantation). **Table 1** summarizes the causes by breaking them down into these categories. In general, the categories can be subdivided by the acuity of the loss of capture, which is usually cardiac in nature. The table also delineates cardiogenic versus noncardiac causes of noncapture in the long-term period postimplant. Reaching the end of the pacemaker or ICD battery can cause loss of capture. At times, reasons for the loss of capture are reversible, but, if the causes cannot be reversed, the lead(s) might need revision/repositioning/replacement or the generator might need to be changed. In preparation for new lead implantation, the pacing mode can be changed to asynchronous pacing at a high output to minimize the chances of noncapture or oversensing noise on a fractured lead. Loss of capture can be detrimental to patients who are dependent upon the pacing function of their device. An acute loss of capture in dependent patients requires hospitalization and either reprogramming of the device at a very high output (often asynchronously) with telemetry monitoring or the insertion of a temporary pacing system until the underlying issue can be resolved emergently.

## Discussion

### Acute loss of capture

**Figure 2:**
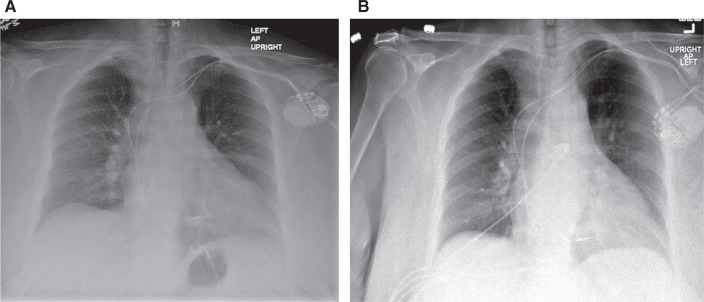
**A:** Chest X-ray at implant with atrial and ventricular leads in place. **B:** Chest X-ray showing atrial lead dislodgment that occurred a few days after device implant.

The most common cause of acute loss of capture after insertion is lead dislodgement or malposition. This can occur within hours to days or even weeks after the procedure. A comparison of the initial chest X-ray and electrocardiogram is usually very helpful. The chest X-ray can reveal the change in location of the lead—unless there is a microdislodgement, which implies micromovement of the lead with no radiographic evidence of the dislodgment.^7^ An example of atrial lead dislodgement on radiographic imaging is shown in **Figure 2**.

In comparison, an electrocardiogram can show a change in the morphology of the captured stimulus if the patient is dependent on pacing or, alternatively, there can be pacing spikes with noncapture in the desired chamber (as shown as **Figure 1**) or capture of a completely different chamber (eg, a dislodged atrial lead can capture ventricular tissue if it has moved past the tricuspid valve). The typical treatment in this case is repositioning of the lead in the postoperative period. Patients who are dependent on pacing may require a temporary pacemaker or asynchronous pacing if there is just an acute increase in the threshold until lead repositioning. Other causes of lead dislodgment including patient factors such as acidemia, ischemia, or acute use of antiarrhythmic agents may appear. However, these are much rarer, given the acuity of the loss of capture within hours to days following implant.

### Long-term causes of loss of capture

An increase in the required threshold leading to a loss of capture can happen after months to years of insertion of the pacemaker or ICD. This can be due to a cardiomyopathy, fibrosis, medications, metabolic imbalance, lead fracture, or an exit block.^5^ Treatment usually involves eliminating or correcting the underlying cause. Until reversal of the underlying factor is achieved, increasing the pacing output can be done to achieve the required threshold. If the patient is dependent on pacing, measures to ensure pacing in the case of an acute loss of capture including temporary pacing or an increase in output to overcome the high threshold until the underlying cause is addressed are necessary.

#### Long-term cardiac causes

Fibrosis and inflammation from the site of lead insertion can cause a loss of capture.^5^ Steroid-eluting tips have decreased the occurrence of fibrosis. Although cardiomyopathy with fibrosis at the site of lead implantation or myocardial infarction at the site of lead implantation can occur, they rarely actually do. If fibrosis or inflammation does occur, repositioning the lead or increasing the output may be helpful adjustments to make. If these areas continue to show fibrosis or infarction despite therapy, lead revision/new implantation may be required depending on the timing of the implant.

Lead failure can present even years after implantation. It is most commonly caused by deterioration of the lead insulation,^8^ although lead failure can also be caused by problems with the connector, simulator electrode, or terminal pin. Extrinsic compression of the lead can also result in failure.^5^ When interrogating the device, a low lead impedance of less than 250 Ω is often seen when the issue concerns the lead insulation.

**Figure 3:**
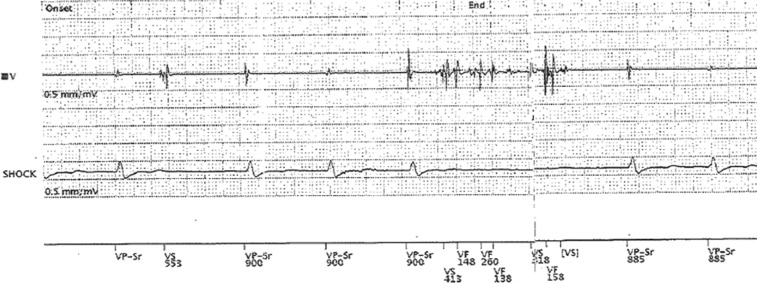
Oversensing of the noise on a ventricular lead in a single-chamber device due to lead fracture as indicated by high-frequency nonphysiologic signals, with a subsequent lack of pacing leading to pauses and syncope.

High-impedance readings can frequently be observed in correlation with lead fracture, even though it is not necessarily present in every case or can be intermittent in nature and not observed during the device interrogation period. Sometimes, the fracture can be visualized on chest X-ray. If lead fracture leads to noncapture, new lead implantation is required, with the urgency of the procedure varying depending on whether the patient has a need for pacing.^9^ **Figure 3** demonstrates noise on a single-chamber ventricular lead from a pacing-dependent patient who experienced lead fracture. The oversensing high-frequency signals due to lead fracture led to a lack of pacing, pauses, and syncope.

Loss of capture can also be attributed to a depletion of battery life. Therefore, it is important to follow up on the life of the battery and to replace the generator when elective replacement is indicated well before to the end of the device’s life. Another cause of noncapture is inappropriate programming of the pacemaker or ICD when there is an insufficient safety margin between the output and threshold values.^2^

#### Long-term noncardiac causes

Electrolyte imbalance and acidosis can cause a loss of capture. Although various electrolyte abnormalities can be correlated with a loss of capture, hyperkalemia is the most common culprit, which usually occurs when the potassium level reaches 7 meq/Ll or higher.^10,11^ Initially, loss of capture can occur due to increased threshold, but, as the level of potassium increases, myocardial conduction is delayed and the paced QRS complex widens. Additionally, when the T-wave starts to increase in amplitude with hyperkalemia, it can be oversensed as a native QRS, leading to a decrease in the frequency of pacing and, ultimately, to bradycardia. Patients who have pacemakers or ICDs who develop hyperkalemia should be managed with reversal of their electrolyte abnormalities immediately, and reprogramming of the cardiac rhythm device may also be needed.^10,11^ Acidemia and hypoxemia can similarly cause a loss of capture. This usually occurs in critically ill patients, and addressing their underlying problems will lead to improvements in the capture threshold.

In rare cases, antiarrhythmic agents can affect the capture threshold significantly and lead to noncapture. Flecainide acetate, a class Ic agent, has been previously associated with a greater-than-200% increase in the capture threshold.^12,13^ The threshold can increase even after one dose of flecainide.^14,15^ Sotalol and amiodarone can also affect the threshold, in that sotalol has been associated with a decrease in defibrillation threshold, whereas amiodarone has a variable effect on the threshold. These are the common antiarrhythmic medications used, but there are many other cardiac medications that can alter the capture threshold as well.^16^ The usual practice of setting an output at a safe margin that is significantly higher than the capture threshold usually prevents an acute loss of capture. If there is a loss of capture in this context, the output can be increased or the antiarrhythmic regimen can be altered to correct the loss of capture.

Finally, external electrical stimulus can be another cause of loss of capture. The source of external stimulus can be misconstrued as ventricular tachycardia/ventricular fibrillation by the pacemaker or ICD, causing asystole depending on the source (as it is sensing an arrhythmia that is not present), and shock therapy can occur as a result in patients with ICDs. This shock therapy can additionally cause an acute rise in the threshold and lead to a temporary loss of capture as well.^17^

## Conclusion

Understanding the cause of loss of capture in pacemakers and ICDs is crucial for the prevention of morbidity, mortality, and inappropriate treatment. There are many causes of a loss of capture, as summarized in **Table 1**. Consideration of the timeline from the implant procedure to the time of the loss of capture is important in determining the cause. It is essential for health-care providers who encounter patients with pacemakers or ICDs to have some understanding of how to correct problems triggering a loss of capture.

## Figures and Tables

**Table 1:** Causes of Loss of Capture

Causes Within Hours to Weeks of Device Implant	Long-term Causes	
	Cardiac	Noncardiac
Lead dislodgment or malposition	Lead fracture	Electrolyte imbalances
Premature lead failure	Fibrosis/inflammation	Acidemia
Premature battery depletion	Cardiomyopathy	Hypoxemia
Programming errors with suboptimal output	Exit block	Medication-induced alterations of the capture threshold
	Breach of insulation	External electrical stimulus
	Battery voltage being at the end of life	
